# Drivers of willingness to communicate with generative AI: the roles of self-efficacy, grit, speaking enjoyment, and anxiety from a self-determination theory perspective

**DOI:** 10.3389/fpsyg.2026.1754495

**Published:** 2026-01-26

**Authors:** Jiaohui Tang, Anchen Zhang, Mingshan Sun, Xinchen Leng, Ling Luo

**Affiliations:** 1 Department of Education, Graduate School, Kookmin University, Seoul, Republic of Korea; 2 Northeastern University, Boston, MA, United States

**Keywords:** anxiety, EFL, enjoyment, generative AI (GenAI), grit, self-determination theory, self-efficacy, willingness to communicate (WTC)

## Abstract

**Introduction:**

English-speaking proficiency is essential for the personal and academic development of English as a Foreign Language (EFL) learners; however, many students demonstrate limited willingness to communicate (WTC) in classroom settings. Although generative artificial intelligence (GenAI) offers considerable potential for enhancing communicative engagement, existing research has predominantly examined WTC in human-to-human interactions, leaving the applicability of established models in AI-mediated environments uncertain.

**Method:**

To address this gap, the present study, guided by self-determination theory, explores the structural relationships among learning grit, speaking self-efficacy, speaking enjoyment, speaking anxiety, and WTC with GenAI. Questionnaire data were collected from 350 Chinese undergraduate EFL learners who practiced oral English using GenAI, and were analyzed using confirmatory factor analysis (CFA) and structural equation modeling (SEM).

**Results and discussion:**

Findings indicate a robust nomological network, with speaking self-efficacy identified as the most influential direct and mediating predictor of WTC with GenAI. Learning grit exerts both direct and indirect effects, while speaking enjoyment and anxiety have comparatively modest impacts. These results suggest that self-efficacy and grit are fundamental psychological drivers of communicative action in GenAI contexts, whereas affective states play a supplementary role. The study extends current WTC frameworks to technology-mediated settings and highlights pedagogical implications for fostering self-belief and perseverance in digital language education.

## 1 Introduction

### 1.1 The role of willingness to communicate in EFL

The primary goal of English as a Foreign Language (EFL) education is to equip learners with the skills necessary for effective and confident communication in real-world contexts, encompassing listening, speaking, reading, and writing (Nation, 2008; Newton and Nation, 2020). Among these four core language skills, speaking is particularly fundamental for daily, academic, and professional interactions, as it constitutes the basis for message delivery, meaning negotiation, social engagement, and personal development (Hargie, 2021; Vygotsky, 1978). Proficiency in spoken English affords EFL learners greater opportunities for accessing quality education and employment, given the status of English as a global lingua franca (Pandey and Pandey, 2014). However, in many countries where English is not the native language, such as China, students often acquire what is referred to as “dumb English”—a phenomenon characterized by the ability to read and write in English without the corresponding ability to speak or comprehend spoken English (Du and Guan, 2016). Examination of this issue has revealed multiple contributing factors, with one central explanation being that many EFL learners lack the willingness to communicate (WTC) in English (Fernández-García and Fonseca-Mora, 2022; Lee and Hsieh, 2019; Rahmatollahi and Khalili, 2015).

Originally conceptualized by MacIntyre et al. (1998) within first language contexts, WTC is defined as an individual’s “readiness to enter into discourse at a particular time with a specific person or persons, using a L2” (MacIntyre et al., 1998, p. 547). A substantial body of empirical research has established that higher levels of WTC are positively correlated with increased frequency of language practice, which, in turn, facilitates greater fluency, enhanced communicative competence, and ultimately, higher levels of language proficiency (e.g., Bergil, 2016; Joe et al., 2017; Mahmoodi and Moazam, 2014). In EFL contexts, WTC represents the crucial psychological threshold that connects learners’ acquired English language knowledge to authentic communicative use. According to the seminal heuristic model proposed by MacIntyre et al. (1998), WTC functions as the immediate antecedent to L2 communicative behavior, shaped by a complex, multi-layered interplay of situational, affective, and motivational factors. Consequently, fostering WTC should be regarded not merely as a desirable educational outcome, but as a fundamental objective for both EFL educators and researchers.

### 1.2 Antecedents and challenges of WTC in EFL

A substantial body of research has examined the factors influencing WTC and has synthesized a network of key psychological constructs that predict L2 WTC in traditional classroom and face-to-face interaction settings (e.g., Riasati and Noordin, 2011; Zhang et al., 2018). These factors can be broadly categorized as situational, motivational, affective, and individual (Li et al., 2025; MacIntyre et al., 1998; MacIntyre et al., 1999; Riasati and Noordin, 2011; Zhang et al., 2018).

Situational antecedents refer to the objective characteristics of a communicative context, such as the interlocutor, the nature of the activity, and the temporal and spatial setting (Kang, 2005; Rauthmann et al., 2015). For example, students may demonstrate greater WTC when interacting with familiar individuals who are perceived as responsive (Pawlak and Mystkowska-Wiertelak, 2015). Similarly, a positive classroom climate has been shown to increase students’ willingness to communicate, whereas a stressful environment tends to inhibit it (Salbaş and Ekmekci, 2025). Motivational antecedents pertain to learners’ drive or readiness to approach or avoid communicative situations (Rauthmann et al., 2015). A salient example is self-efficacy in speaking—the belief in one’s ability to successfully execute a speaking task—which has emerged as a primary motivational force (Leeming et al., 2024). Learners with high self-efficacy are more likely to perceive communicative challenges as opportunities to be mastered, rather than threats to be avoided. Affective factors involve emotional states that influence both WTC and actual communicative performance (Lee and Hsieh, 2019). Speaking anxiety, for instance, is a powerful negative predictor of WTC, often creating psychological barriers that inhibit learners from participating, even when they possess the necessary linguistic skills (Zhou et al., 2023). Conversely, speaking enjoyment—a positive emotional experience—has been shown to enhance learners’ willingness to engage in communication and to take communicative risks (Lee, 2022). Individual, or trait-like, dispositions refer to relatively stable personal characteristics such as personality, gender, and age, which shape learners’ WTC. Notably, the construct of grit, defined as perseverance and passion for long-term goals (Duckworth et al., 2007), has been found to support sustained effort and motivation in the face of the inevitable challenges of language learning, thereby contributing to the maintenance of WTC over time (Chen et al., 2025; Lee, 2022).

Despite this nuanced understanding of WTC predictors, a significant practical challenge persists in many EFL contexts: the “Practice Paradox.” Learners require a safe, low-anxiety environment to practice speaking regularly in order to build communicative skills and confidence. However, the traditional classroom setting—characterized by social evaluation and fear of losing face in front of peers and teachers—often becomes a source of anxiety that directly inhibits communicative practice (Aziz and Kashinathan, 2021; Maher and King, 2023; Suleimenova, 2013). This dynamic creates a self-perpetuating cycle: anxiety diminishes WTC, resulting in reduced practice, which in turn leads to lower proficiency and heightened anxiety. While pedagogical interventions such as cooperative learning and task-based language teaching have been proposed as potential solutions (e.g., Bárkányi and Brash, 2025), these approaches frequently struggle to provide every learner with ample, individualized, and genuinely anxiety-free speaking opportunities.

### 1.3 Generative AI as a potential solution to improve WTC

The advent of sophisticated generative artificial intelligence (GenAI) chatbots, such as ChatGPT by OpenAI and Doubao by Chinese TikTok, represents a transformative opportunity to disrupt existing patterns in EFL learning. GenAI systems, particularly those based on large language models (LLMs), function as perpetually accessible, infinitely patient, and non-judgmental conversational partners. These models are trained on extensive corpora using reinforcement learning from human feedback (Guo and Wang, 2024), enabling them to generate responses that are human-like, safe, and non-harmful. When integrated with text-to-speech technology, GenAI chatbots can interact with learners using naturalistic voice and tone, further enhancing the authenticity of the conversational experience (Celik et al., 2025). Consequently, GenAI exhibits significant potential to revolutionize students’ speaking practice through its unique affordances, which may be theorized to address known inhibitors of willingness to communicate (WTC).

From an individual trait perspective, GenAI offers students unrestricted access at any time and from any location. This accessibility allows learners with high learning grit to consistently engage in practice, unimpeded by traditional constraints such as classroom schedules or the availability of partners. From an affective perspective, the novelty, interactivity, and personalized nature of GenAI-mediated conversations can increase learner engagement and enjoyment, thereby enhancing students’ speaking enjoyment. Additionally, GenAI provides a psychologically safe environment that removes the fear of negative social evaluation, theoretically reducing speaking anxiety and enabling learners to experiment with language without apprehension. From a motivational perspective, GenAI facilitates repeated practice opportunities, allowing learners to control conversation topics, complexity, and pace, which can lead to mastery experiences and foster greater speaking self-efficacy.

Therefore, GenAI should not be regarded merely as a technological tool; rather, it constitutes a fundamentally new type of interlocutor with the potential to reconfigure the psychological ecosystem in which English as a Foreign Language (EFL) speaking practice occurs.

Any alternative text (alt text) provided alongside figures in this article has been generated by Frontiers with the support of artificial intelligence and reasonable efforts have been made to ensure accuracy, including review by the authors wherever possible. If you identify any issues, please contact us.

### 1.4 Underpinning theory and research questions

Despite the intuitively appealing potential of GenAI, a critical question remains regarding how its unique affordances reshape the psychology of language learning. Existing research on WTC is predominantly grounded in human-to-human interaction (e.g., Peng and Woodrow, 2010; Wang et al., 2023), where social dynamics are central. However, GenAI introduces a fundamentally different ecological niche characterized by low social-evaluative threat, high accessibility, and non-anthropomorphic interactions. Consequently, the research gap is not merely whether canonical WTC models hold in this new setting, but whether the strength and sequence of psychological paths are reconfigured by these specific contextual features. For instance, in an environment devoid of human judgment, does the inhibitory path of speaking anxiety weaken? Does the constant availability of GenAI strengthen the predictive power of learning grit? Currently, the field lacks a theoretical understanding of how these established variables interact within the specific “safe haven” of GenAI-mediated communication.

To address these complex relationships, this study adopts Self-Determination Theory (SDT; Deci and Ryan, 2012; Ryan and Deci, 2002) as its overarching theoretical framework. SDT posits that optimal motivation and engaged behavior occur in environments that support three basic psychological needs: competence, autonomy, and relatedness.

In the specific context of GenAI-mediated language learning, we conceptualize these needs as being supported—or thwarted—by learners’ individual traits (grit), motivational beliefs (self-efficacy), and affective states (anxiety and enjoyment). First, the need for competence refers to the need to feel effective in one’s interactions. In this study, speaking self-efficacy acts as the primary psychological indicator of competence satisfaction. As noted by Bandura (1997), self-efficacy reflects a learner’s judgment of their capabilities; when interacting with GenAI, a learner with high self-efficacy perceives themselves as capable of handling the digital interlocutor, thereby fulfilling the need for competence in this specific task domain. Second, the need for autonomy concerns the feeling of volition and self-direction in one’s actions. While GenAI offers the opportunity for autonomous practice (e.g., choice of topic and pace), learning grit represents the internal capacity to sustain this autonomy. Grit, defined as sustained passion and perseverance for personally meaningful goals, serves as a form of autonomous self-regulation (Duckworth et al., 2007). It allows learners to maintain self-directed engagement with GenAI despite the lack of external teacher supervision, thus operationalizing autonomy in a solitary learning context. Third, and perhaps most critical in non-human interaction, is the need for relatedness, which typically involves feeling connected to others. We posit that while GenAI cannot offer human intimacy, it satisfies a functional form of relatedness by providing psychological safety and a pseudo-social presence. However, we explicitly acknowledge that this is a context-specific operationalization rather than a complete conceptual equivalence to human relatedness. While interaction with an algorithm does not provide the deep interpersonal bonding found in human relationships, it captures the critical “security” dimension of relatedness by minimizing social-evaluative threat. In traditional classrooms, relatedness is often threatened by social anxiety and fear of judgment. GenAI mitigates this threat. Therefore, within this specific digital ecology, low speaking anxiety and high speaking enjoyment serve as proxies for relatedness satisfaction in this context. The absence of anxiety signals a safe, non-judgmental environment (a “safe haven”), while high enjoyment indicates a positive emotional connection to the interaction (Lee and Hsieh, 2019). Together, these affective states foster a sense of being accepted by the interface, effectively reconfiguring the need for relatedness from social bonding to psychological security.

### 1.5 Research questions

Based on this theoretical foundation, we first posit that these autonomy-, competence-, and relatedness-supportive variables directly drive learners’ WTC with GenAI. Specifically, we hypothesize that the positive affective state of enjoyment fuels the desire to interact, while residual anxiety may still inhibit it (MacIntyre et al., 1998). Simultaneously, high self-efficacy (competence) encourages learners to approach communicative challenges (Peng and Woodrow, 2010), and grit (autonomy) provides the sustained drive to initiate practice. Thus, we propose: speaking anxiety negatively predicts WTC with GenAI (H4); speaking enjoyment positively predicts WTC with GenAI (H5); speaking self-efficacy positively predicts WTC with GenAI (H6); and learning grit positively predicts WTC with GenAI (H7).

**Figure 1:**
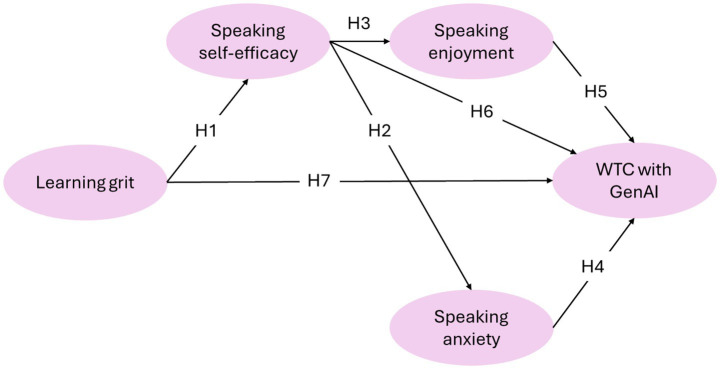
Hypothesized structural equation model for learning grit, speaking self-efficacy, speaking enjoyment, speaking anxiety, and WTC with GenAI.

Beyond these direct associations, we further propose a structural sequence where distal traits influence proximal states, forming the internal logic of the model (Figure 1). First, we argue that grit facilitates self-efficacy. Gritty learners are more likely to persist in practice, accumulating the mastery experiences that Bandura (1997) identifies as the primary source of efficacy. Thus, we hypothesize that learning grit positively predicts speaking self-efficacy (H1). Second, we argue that self-efficacy shapes affective states. When learners feel efficacious, they appraise the GenAI interaction as manageable, which reduces fear and enhances pleasure (Teimouri et al., 2019). Thus, we hypothesize that speaking self-efficacy negatively predicts speaking anxiety (H2) and positively predicts speaking enjoyment (H3).

Integrating these hypotheses, the proposed model (Figure 1) examines both the direct and indirect pathways influencing WTC. This study, therefore, aims to address the following research questions (RQs):

- RQ1: Is there a significant relationship between EFL students’ learning grit, speaking self-efficacy, speaking enjoyment, speaking anxiety, and WTC with GenAI?
- RQ2: To what extent do EFL students’ learning grit, speaking self-efficacy, speaking enjoyment, and speaking anxiety predict their WTC with GenAI?

## 2 Method

### 2.1 Participants

The participants in this study comprised 350 Chinese undergraduate students with prior experience using GenAI for English speaking practice. The sample included 176 females (50.3%) and 174 males (49.7%). Participants were recruited from multiple universities in northwestern China in collaboration with local researchers. Undergraduate EFL learners were specifically targeted, as proficiency in spoken English is essential for pursuing advanced degrees (e.g., studying abroad for master’s or doctoral programs) and for seeking employment in the global job market. The sample included students from all four undergraduate years: 74 first-year students (21.1%), 104 s-year students (29.7%), 103 third-year students (29.4%), and 69 fourth-year students (19.7%). Participants’ ages ranged from 18 to 22 years (M = 19.9). To characterize the sample’s linguistic profile, English proficiency was assessed using College English Test Band 4 (CET-4) scores, a standardized national English test in China. The majority of participants possessed intermediate to upper-intermediate proficiency: 33.7% scored between 550 and 600, 24.6% between 500 and 550, and 22.0% between 425 and 500. A smaller proportion scored above 600 (18.0%) or below 425 (1.7%). Regarding GenAI experience, all participants reported using “Doubao” (a popular GenAI tool by ByteDance) for oral practice. In terms of usage frequency, the majority were moderate users, with 59.1% practicing 3–5 times per month, followed by 26.6% utilizing it less than 3 times per month, and 14.3% engaging with the tool more than 5 times per month. Detailed demographic information is presented in Table 1.

**Table 1:** Demographic information of the participants (*n* = 350).

Variable	Category	Frequency	Percentage
Gender	Female	176	50.3%
	Male	174	49.7%
Age	18	57	16.3%
	19	85	24.3%
	20	93	26.6%
	21	64	18.3%
	22	51	14.6%
Grade	Year 1	74	21.1%
	Year 2	104	29.7%
	Year 3	103	29.4%
	Year 4	69	19.7%
CET-4	< 425	6	1.7%
	425–500	77	22.0%
	500–550	86	24.6%
	550–600	118	33.7%
	>600	63	18.0%
GenAI usage for practicing oral English	< 3 times per month	93	26.6%
	3–5 times per month	207	59.1%
	>5 times per month	50	14.3%

### 2.2 Instruments

#### 2.2.1 Learning grit

To assess students’ grit in the context of EFL, this study employed the L2-Grit Scale developed and validated by Alamer (2021). This instrument is specifically designed to measure learners’ trait-level perseverance and sustained passion for second language learning, making it well-suited to the present research context. The scale utilized consists of six items, such as “I often set a language learning goal but later choose to pursue a different one” (reverse-coded), which evaluate an individual’s ability to maintain consistent focus and long-term commitment to learning English, rather than being distracted by new interests (Alamer, 2021). Participants responded to each item using a 5-point Likert scale ranging from 1 (Strongly Disagree) to 5 (Strongly Agree). The construct validity of the scale was rigorously established by Alamer (2021) through both exploratory and confirmatory factor analyses. Additionally, the scale demonstrated excellent reliability, with a Cronbach’s alpha of 0.908.

#### 2.2.2 Speaking self-efficacy

To evaluate students’ self-efficacy in English speaking, this study utilized the scale developed and validated by Leeming (2017) and Leeming et al. (2024), which specifically examines EFL learners’ speaking efficacy. In accordance with Bandura’s (1997) recommendation that questionnaire items should be directly related to the task, the scale was designed to reflect various aspects of speaking practice, encompassing a range of competencies and skills such as expressing opinions and asking follow-up questions (Leeming et al., 2024). The instrument consists of 10 items, each rated on a 5-point Likert scale. The construct validity of the scale has been rigorously established (Leeming et al., 2024), and it demonstrates excellent reliability, with a Cronbach’s alpha of 0.941.

#### 2.2.3 Speaking enjoyment

To assess students’ enjoyment specific to EFL speaking with GenAI, this study adapted the English speaking enjoyment scale originally developed and validated by Jin and Qin (2024). This scale was specifically designed to evaluate the enjoyment experienced by Chinese university students when speaking English, making it highly relevant to the present research context. The instrument comprises 12 items that measure both enduring manifestations of speaking enjoyment, such as affective states and a sense of accomplishment, as well as the dynamic and expressive aspects of enjoyment, including changes in facial expressions and emotions during speaking (Jin and Qin, 2024). For this study, the scale was modified by replacing “English speaking” with “English speaking with GenAI” to maintain contextual relevance without altering the core construct. The adapted scale was reviewed by experts in English speaking enjoyment to ensure content validity. Each item was rated on a 5-point Likert scale. Construct validity was further established through confirmatory factor analysis (CFA), with results indicating X^2^ = 51.804, df = 54, X^2^/df = 0.959, *p* > 0.05, CFI > 0.95, TLI > 0.95, RMSEA < 0.08, and SRMR = 0.018, all of which suggest strong construct validity. Additionally, the scale demonstrated excellent reliability, with a Cronbach’s alpha of 0.945.

#### 2.2.4 Speaking anxiety

To assess students’ anxiety specific to EFL speaking with GenAI, this study adapted the validated short-form foreign language classroom anxiety scale (Botes et al., 2022). The original scale was designed to measure speaking anxiety in a foreign language classroom setting. For the present study, the items were contextually modified to reflect the unique environment of speaking practice with GenAI tools. Specifically, references to the general “foreign language class” were replaced with phrases such as “speaking English with GenAI” or “using GenAI for spoken English practice.” For example, the original item “It embarrasses me to volunteer answers in my FL class” was adapted to “It embarrasses me to speak English when using GenAI tools.” This adaptation ensured that the scale continued to measure the core construct of speaking anxiety while aligning with the study’s context. The adapted scale was reviewed by experts in English speaking anxiety to ensure content validity. The final version retained the original 8-item structure and utilized a 5-point Likert response format. Construct validity was further confirmed through confirmatory factor analysis (CFA), with results indicating X^2^ = 26.607, df = 21, X^2^/df = 1.267, p > 0.05, CFI > 0.95, TLI > 0.95, RMSEA < 0.08, and SRMR = 0.017, all supporting strong construct validity. Additionally, the scale demonstrated excellent reliability, with a Cronbach’s alpha of 0.927.

#### 2.2.5 WTC with GenAI

To assess students’ WTC with GenAI, this study adapted the validated WTC scale developed by Zhang et al. (2024). The original scale was designed to measure Chinese university students’ WTC with AI, specifically referring to the tool iFLYTEK Spark. For the present study, the items were contextually modified to reflect the environment of speaking practice with GenAI tools by replacing references to “iFLYTEK Spark” with “GenAI.” For example, the original item “I think communicating with iFLYTEK Spark develops my speaking fluency” was adapted to “I think communicating with GenAI develops my speaking fluency.” This adaptation ensured the scale continued to measure the core construct of willingness to communicate while aligning with the specific context of this research. The adapted scale was reviewed by experts in WTC to ensure content validity. The final instrument consisted of ten items, each rated on a 5-point Likert scale. Construct validity was further confirmed through confirmatory factor analysis (CFA), with results indicating X^2^ = 90.218, df = 36, X^2^/df = 2.506, *p* < 0.001, CFI > 0.95, TLI > 0.95, RMSEA < 0.08, and SRMR = 0.056, all of which support strong construct validity. Additionally, the scale demonstrated excellent reliability, with a Cronbach’s alpha of 0.929.

### 2.3 Data collection and analysis

Data collection was facilitated by the participants’ teachers through a standardized two-step recruitment process to ensure sample validity. First, during classroom sessions, instructors screened students to identify those who had prior experience using GenAI for English speaking practice. Only eligible students were then invited to join a specific WeChat group where the survey link was distributed. The questionnaire was hosted on Wenjuanxing—a widely used Chinese survey platform. It required participants to report on their learning grit, speaking self-efficacy, speaking enjoyment, speaking anxiety, and WTC with GenAI, as well as to provide personal background information. Completion of the survey took approximately 10–15 min. Participation was voluntary, and all respondents were informed that their information would remain confidential and be used solely for research purposes.

For data analysis, the data underwent a rigorous quantitative processing pipeline prior to hypothesis testing. First, raw data were screened for missing values and outliers. No missing data were identified as the online platform mandated responses for all items. Multivariate normality was assessed by examining skewness and kurtosis values, which fell within the acceptable range recommended by Kline (2023). Given that the data relied on self-reported measures collected at a single time point, Harman’s single-factor test was conducted to check for potential common method bias (Podsakoff et al., 2003). The results showed that the first factor accounted for 33.27% of the total variance, which is well below the 50% threshold, indicating that common method bias was not a significant concern. Subsequently, CFA was conducted to evaluate the measurement model encompassing all key constructs: learning grit, speaking self-efficacy, speaking enjoyment, speaking anxiety, and WTC with GenAI. The relationships among these variables (RQ1) were examined through the results of the CFA. To address the extent to which learning grit, speaking self-efficacy, speaking enjoyment, and speaking anxiety predict EFL students’ WTC with GenAI (RQ2), structural equation modeling (SEM) was employed. All statistical analyses were performed using SPSS 31 and AMOS 29.

## 3 Results

### 3.1 CFA results

CFA was conducted to assess the measurement model. The model demonstrated an excellent fit to the data: X^2^ = 1159.797, df = 984, X^2^/df = 1.179, CFI = 0.984, TLI = 0.983, RMSEA = 0.023, SRMR = 0.037. All fit indices met or exceeded the established thresholds for excellent model fit (Hu and Bentler, 1999), with χ^2^/df < 3, CFI > 0.95, TLI > 0.95, RMSEA < 0.06, and SRMR < 0.08.

As presented in Table 2, all factor loadings were statistically significant (*p* < 0.001) and substantial in magnitude, with standardized estimates exceeding the recommended value of 0.6 (Hair et al., 2010). This indicates that all items were robust indicators of their respective latent constructs.

**Table 2:** Unstandardized and standardized estimates in the CFA model.

Relation	Standardized estimates	Unstandardized estimates	S. E.	C. R.	*p*
Grit1 ← Learning grit	0.774	0.956	0.062	15.341	***
Grit2 ← Learning grit	0.793	1.039	0.065	16.029	***
Grit3 ← Learning grit	0.812	0.998	0.061	16.32	***
Grit4 ← Learning grit	0.774	1.007	0.065	15.379	***
Grit5 ← Learning grit	0.798	1.005	0.062	16.096	***
Grit6 ← Learning grit	0.786	1			
Efficacy1 ← Speaking self-efficacy	0.766	1			
Efficacy2 ← Speaking self-efficacy	0.78	1			
Efficacy3 ← Speaking self-efficacy	0.792	1.064	0.051	20.727	***
Efficacy4 ← Speaking self-efficacy	0.791	1.038	0.05	20.703	***
Efficacy5 ← Speaking self-efficacy	0.799	1.047	0.05	21.079	***
Efficacy6 ← Speaking self-efficacy	0.785	1.033	0.051	20.365	***
Efficacy7 ← Speaking self-efficacy	0.778	0.985	0.049	20.006	***
Efficacy8 ← Speaking self-efficacy	0.793	1.016	0.049	20.755	***
Efficacy9 ← Speaking self-efficacy	0.776	1			
Efficacy10 ← Speaking self-efficacy	0.793	1			
Anxiety1 ← Speaking anxiety	0.774	1			
Anxiety2 ← Speaking anxiety	0.794	0.982	0.052	18.758	***
Anxiety3 ← Speaking anxiety	0.763	0.983	0.056	17.53	***
Anxiety4 ← Speaking anxiety	0.783	0.959	0.053	18.246	***
Anxiety5 ← Speaking anxiety	0.788	0.98	0.053	18.54	***
Anxiety6 ← Speaking anxiety	0.777	0.968	0.053	18.107	***
Anxiety7 ← Speaking anxiety	0.809	1.029	0.053	19.428	***
Anxiety8 ← Speaking anxiety	0.788	1			
Enjoy1 ← Speaking enjoyment	0.746	1			
Enjoy2 ← Speaking enjoyment	0.794	1.105	0.072	15.358	***
Enjoy3 ← Speaking enjoyment	0.786	1.059	0.07	15.179	***
Enjoy4 ← Speaking enjoyment	0.788	1.044	0.068	15.298	***
Enjoy5 ← Speaking enjoyment	0.79	1.082	0.071	15.272	***
Enjoy6 ← Speaking enjoyment	0.761	1.013	0.069	14.583	***
Enjoy7 ← Speaking enjoyment	0.796	1.061	0.069	15.398	***
Enjoy8 ← Speaking enjoyment	0.757	0.976	0.067	14.567	***
Enjoy9 ← Speaking enjoyment	0.734	0.925	0.066	14.103	***
Enjoy10 ← Speaking enjoyment	0.751	0.958	0.067	14.393	***
Enjoy11 ← Speaking enjoyment	0.744	0.91	0.063	14.358	***
Enjoy12 ← Speaking enjoyment	0.766	1.031	0.07	14.728	***
WTC1 ← WTC	0.606	1			
WTC2 ← WTC	0.768	1.113	0.072	15.47	***
WTC3 ← WTC	0.787	1.146	0.071	16.06	***
WTC4 ← WTC	0.811	1.167	0.069	16.793	***
WTC5 ← WTC	0.789	1.154	0.072	16.048	***
WTC6 ← WTC	0.789	1.192	0.074	16.101	***
WTC7 ← WTC	0.772	1.115	0.071	15.71	***
WTC8 ← WTC	0.788	1.157	0.072	16.112	***
WTC9 ← WTC	0.734	1			
WTC10 ← WTC	0.784	1.144	0.072	15.989	***

**p < 0.01.

The composite reliability (CR) and convergent validity of the constructs were also examined. As shown in Table 3, the CR values for WTC with GenAI, speaking enjoyment, speaking anxiety, speaking self-efficacy, and learning grit were 0.933, 0.945, 0.928, 0.941, and 0.909, respectively. All values surpassed the recommended threshold of 0.70, indicating satisfactory internal consistency (Hair et al., 2010). Furthermore, the average variance extracted (AVE) for these constructs were 0.585, 0.590, 0.616, 0.617, and 0.623, all of which exceeded the benchmark of 0.50. This provides evidence of convergent validity, confirming that the constructs adequately explain the variance of their indicators (Hair et al., 2010). Additionally, the assessment of discriminant validity confirms that the constructs are distinct from one another. The square root of the AVE for each construct, displayed on the diagonal, is greater than its correlations with all other constructs, thereby satisfying the Fornell-Larcker criterion.

**Table 3:** Reliability and validity of the variables, as well as their correlation.

Variable	CR	AVE	WTC with GenAI	Speaking enjoyment	Speaking anxiety	Speaking self-efficacy	Learning grit
WTC with GenAI	0.933	0.585	0.765				
Speaking enjoyment	0.945	0.590	0.423***	0.768			
Speaking anxiety	0.928	0.616	−0.393***	−0.398***	0.785		
Speaking self-efficacy	0.941	0.617	0.463***	0.449***	−0.41***	0.785	
Learning grit	0.909	0.623	0.466***	0.491***	−0.439***	0.438***	0.790

**p < 0.01.

### 3.2 Variable relationship

The correlation matrix, as presented in the right part of Table 3, further reveals a conceptually coherent pattern of relationships. WTC with GenAI demonstrates significant positive correlations with speaking enjoyment (*r* = 0.423, *p* < 0.001), speaking self-efficacy (*r* = 0.463, *p* < 0.001), and learning grit (*r* = 0.466, *p* < 0.001). Conversely, WTC with GenAI is negatively correlated with speaking anxiety (*r* = −0.393, *p* < 0.001). This pattern of a positive network among the adaptive constructs (enjoyment, self-efficacy, and grit) and their shared inverse relationship with speaking anxiety supports the theoretical nomological network, indicating that higher levels of positive psychological attributes and lower anxiety are associated with a greater willingness to use GenAI for communication.

### 3.3 SEM results

**Figure 2:**
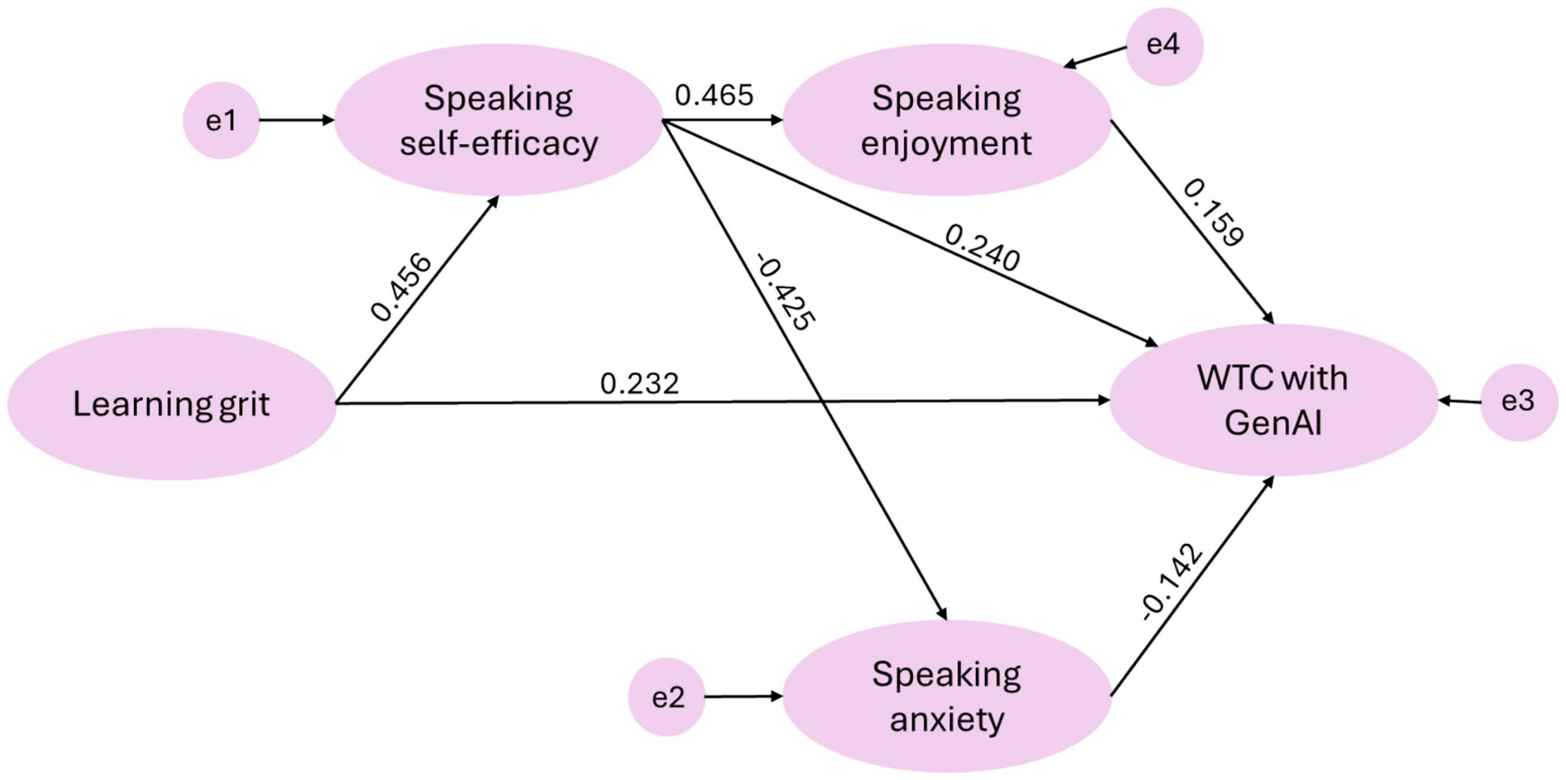
Structural equation model with standardized regression weights.

The SEM analysis shows an excellent fit of the model proposed in Figure 1: X^2^ = 1236.897, df = 987, X^2^/df = 1.253, CFI = 0.977, TLI = 0.976, RMSEA = 0.027, SRMR = 0.079. All fit indices met or exceeded the established thresholds for excellent model fit (Hu and Bentler, 1999), with χ^2^/df < 3, CFI > 0.95, TLI > 0.95, RMSEA < 0.06, and SRMR < 0.08. Table 4 and Figure 2 show the regression weights of the SEM analysis. The model indicates that all four hypothesized factors—learning grit, speaking self-efficacy, speaking enjoyment, and speaking anxiety—are statistically significant direct predictors of WTC with GenAI, collectively outlining a clear psychological profile of students inclined to engage with AI for language practice.

**Table 4:** Results of regression analysis with SEM.

Hypotheses	Pathway	Standardized	Unstandardized				Supported (Yes/No)
		Estimates	Estimates	S. E.	C. R.	*p*	
H1	Speaking self-efficacy ← Learning grit	0.456	0.426	0.053	8.088	***	Yes
H2	Speaking anxiety ← Speaking self-efficacy	−0.425	−0.384	0.049	−7.794	***	Yes
H3	Speaking enjoyment ← Speaking self-efficacy	0.465	0.395	0.048	8.224	***	Yes
H4	WTC with GenAI ← Speaking anxiety	−0.142	−0.119	0.049	−2.451	*	Yes
H5	WTC with GenAI ← Speaking enjoyment	0.159	0.142	0.053	2.651	*	Yes
H6	WTC with GenAI ← Speaking self-efficacy	0.240	0.181	0.047	3.875	***	Yes
H7	WTC with GenAI ← Learning grit	0.232	0.164	0.044	3.708	***	Yes

*p < 0.05, ***p < 0.001.

Speaking self-efficacy emerged as the strongest positive direct predictor of WTC with GenAI (*β* = 0.240, *p* < 0.001), suggesting that students with greater confidence in their speaking abilities are more likely to use GenAI for communication. Learning grit also demonstrated a substantial and significant direct effect (*β* = 0.232, *p* < 0.001), indicating that students who are more persistent and passionate about long-term goals are more willing to engage with this technology. Furthermore, the model reveals significant, though comparatively weaker, direct effects from speaking enjoyment (*β* = 0.159, *p* < 0.05) and speaking anxiety (*β* = −0.142, *p* < 0.05). This indicates that while a positive emotional experience with speaking fosters WTC, anxiety exerts a distinct, dampening effect, even when controlling for other factors.

Beyond these direct effects, the path coefficients suggest a more complex nomological network. Learning grit exhibits a strong positive relationship with speaking self-efficacy (*β* = 0.456, *p* < 0.001), which in turn is a powerful antecedent to both speaking enjoyment (*β* = 0.465, *p* < 0.001) and speaking anxiety (*β* = −0.425, *p* < 0.001). This implies that the influence of grit on WTC with GenAI is partially mediated through its enhancement of self-efficacy and the subsequent improvement in affective states. Specifically, the analysis of standardized indirect effects reveals that learning grit exerts a robust indirect influence on WTC (*β* = 0.170), which is nearly comparable in magnitude to its direct effect. Similarly, speaking self-efficacy exerts a meaningful indirect effect (*β* = 0.134) through the regulation of anxiety and enjoyment, indicating that a substantial portion of its impact is channeled through these affective mediators. Consequently, the total effect of learning grit and speaking self-efficacy on WTC is likely amplified through these direct and indirect pathways, positioning self-efficacy as a central hub that transmits the influence of grit and regulates emotional responses, ultimately shaping a student’s decision to communicate with GenAI.

## 4 Discussion and conclusion

Despite the pivotal role of English-speaking proficiency in the personal development of EFL learners, a significant proportion of EFL students exhibit limited WTC within classroom environments. With advancements in technology, GenAI shows considerable promise in enhancing EFL learners’ WTC by influencing a range of psychological factors. Nevertheless, existing research on WTC has predominantly concentrated on human-to-human interaction, leaving it unclear whether established structural relationships are equally applicable when GenAI serves as the interlocutor. Accordingly, this study aims to examine the intricate interplay of psychological variables that predict EFL students’ WTC in interactions with GenAI.

Drawing on self-determination theory, this research pursues two primary objectives: (1) to examine the relationships among learning grit, speaking self-efficacy, speaking enjoyment, speaking anxiety, and WTC with GenAI; and (2) to quantify the predictive power and structural interrelations among these constructs. The study utilized CFA and SEM to analyze survey data collected from 350 Chinese undergraduate EFL learners who engaged with GenAI for oral English practice, thereby providing robust and nuanced insights into these questions.

### 4.1 Findings

The findings collectively indicate that learning grit, speaking self-efficacy, speaking enjoyment, speaking anxiety, and WTC with GenAI constitute a tightly interconnected and statistically significant nomological network. As presented in Table 3, the correlation matrix illustrates a conceptually coherent pattern: WTC with GenAI is positively correlated with speaking enjoyment (*r* = 0.423, *p* < 0.001), speaking self-efficacy (*r* = 0.463, *p* < 0.001), and learning grit (*r* = 0.466, *p* < 0.001), and negatively correlated with speaking anxiety (*r* = −0.393, *p* < 0.001). This pattern is highly consistent with the central tenets of self-determination theory (Deci and Ryan, 2012; Ryan and Deci, 2002), which posits that fulfillment of three fundamental psychological needs—autonomy, competence, and relatedness—is essential for fostering high-quality motivation and engagement.

The positive associations between WTC with GenAI and the adaptive constructs can be interpreted within this theoretical framework. Speaking self-efficacy directly reflects the need for competence; students who are confident in their English-speaking abilities are more likely to perceive interactions with GenAI as manageable and successful, thereby helping satisfy their competence need and enhance their WTC. Speaking enjoyment pertains to the need for relatedness, as enjoyment is often derived from engaging in intrinsically interesting activities, thus fostering a sense of connection between learners and GenAI. The positive correlation indicates that students who experience enjoyment in speaking are more motivated to pursue opportunities for interaction, such as those facilitated by GenAI. Furthermore, learning grit—defined as passion and perseverance toward long-term goals—can be conceptualized as self-concordance and integrated regulation, representing advanced forms of autonomous motivation in self-determination theory (Deci and Ryan, 2012; Ryan and Deci, 2002). Gritty students are driven by deeply internalized values, prompting them to utilize all available resources, including GenAI, to achieve their learning objectives.

The negative correlation between speaking anxiety and WTC with GenAI aligns with previous research on traditional WTC (e.g., Ghani and Azhar, 2017; Zarrinabadi et al., 2023) and extends these findings to the GenAI context. Anxiety may impede the need for competence by engendering feelings of inadequacy and fear of negative evaluation. Although evaluation from AI may be perceived as less threatening than human evaluation, our results suggest that anxiety remains a significant psychological barrier to communication, even in technologically mediated environments.

The SEM results, presented in Table 4 and Figure 2 and Table A1, advance the analysis beyond simple bivariate relationships by elucidating a sophisticated model of direct and indirect effects, thereby shedding light on the potential underlying mechanisms. Notably, speaking self-efficacy emerged as the most influential direct positive predictor of WTC with GenAI (*β* = 0.240, *p* < 0.001) and functioned as a central mediator within the model. Significant pathways from learning grit to self-efficacy (*β* = 0.456, p < 0.001), and from self-efficacy to both speaking enjoyment (β = 0.465, p < 0.001) and speaking anxiety, reveal pronounced indirect effects. This suggests that students’ grit is associated with enhanced self-efficacy, which subsequently relates to increased speaking enjoyment and diminished speaking anxiety (*β* = −0.425, *p* < 0.001), corroborating previous findings (e.g., Chen, 2025; Wang and Wang, 2025; Zhang et al., 2024; Zhang et al., 2025). These affective states, in turn, influence WTC with GenAI, establishing potential directional pathway supported by the model: Grit → Self-Efficacy → (Enjoyment/Anxiety) → WTC with GenAI. However, given the cross-sectional design, this sequence should be interpreted as a theoretical structure rather than definitive temporal causality.

This outcome resonates with Bandura’s (2001) social cognitive theory, which identifies self-efficacy as a primary determinant of behavior, and with self-determination theory, wherein perceived competence is foundational. The findings indicate that self-efficacy is not merely a correlate but a fundamental psychological resource, serving to link sustained effort—characteristic of grit—to positive affective states and, ultimately, communicative action (Fathi et al., 2021). This dual pathway helps explain why self-efficacy exerts the largest total effect on WTC with GenAI, with its influence both direct and mediated via multiple affective mechanisms.

The direct effects of learning grit further underscore its significance. Even after accounting for its mediation through self-efficacy, the direct path remains significant (*β* = 0.232, *p* < 0.001), suggesting that gritty students possess a resilient, goal-oriented disposition that motivates them to utilize GenAI regardless of transient fluctuations in confidence or anxiety. Such students may perceive GenAI as a strategic tool for long-term mastery, consistent with the concept of “purposeful perseverance” (Duckworth et al., 2007). This dual function of grit—as both a direct predictor and an indirect facilitator via self-efficacy—highlights its status as a critical non-cognitive foundation for adaptive learning behaviors in digital learning contexts.

The direct effects of affective variables, specifically speaking enjoyment (*β* = 0.159, *p* < 0.05) and speaking anxiety (*β* = −0.142, *p* < 0.05), while statistically significant, were comparatively modest in magnitude. This suggests that although emotional states are significantly associated with WTC, their direct impact is less pronounced than the foundational influences of self-belief and grit. We speculate that this may represent a unique feature of the GenAI context, in which the private, non-judgmental nature of GenAI interaction likely mitigates the typically powerful impact of anxiety observed in human-to-human communication (Wu et al., 2025). Similarly, the intrinsic enjoyment derived from the activity may be somewhat less pivotal if GenAI is perceived primarily as a pragmatic learning tool rather than a purely social partner. While our data do not directly measure learners’ role perceptions of GenAI, this inference aligns with the observed dominance of competence-related variables.

### 4.2 Implications, limitations, and future research

#### 4.2.1 Theoretical implications

The findings of this study offer implications that go beyond merely validating existing models; rather, they suggest a re-specification of L2 WTC theory for the emerging context of de-humanized environments. Traditionally, WTC models rely heavily on social relatedness and the management of interpersonal anxiety. However, this study reveals that in the depersonalized learning space of human–GenAI interaction, the removal of human interlocutors appears to shift the drivers of communication. As the social-evaluative threat likely diminishes, competence (speaking self-efficacy) and autonomous regulation (learning grit) gain significantly greater explanatory weight. This re-specification implies that GenAI-mediated WTC may be driven less by the need for social bonding and more by an internal drive for mastery and self-regulation. The structural model supports integrating SDT with social cognitive theory to explain this shift: specifically, the fulfillment of the competence need (self-efficacy), catalyzed by the trait-level autonomy of grit, seems to act as a central engine regulating emotional experiences. Consequently, affective antecedents appear to follow altered pathways—anxiety may act not as a social barrier but as a performance regulator, while enjoyment is derived from the safe exercise of agency. This offers a new theoretical lens on motivation-behavior links, positing that in non-human interactions, the “self” (efficacy and grit) may supersede the “social” as the primary architect of willingness to communicate.

#### 4.2.2 Practical implications

Based on these findings, this study provides actionable pedagogical strategies for EFL instructors to enhance student engagement with GenAI. First, given that speaking self-efficacy emerged as the strongest predictor, instructional designs would benefit from prioritizing mastery experiences through graduated complexity. Instructors are encouraged to design a graduated GenAI speaking-task sequence. For instance, students could begin with a low-stakes 1-min self-introduction, progress to a 3-min simulated interview, and culminate in a complex 5-min debate with the GenAI agent. To further reinforce competence, explicit instruction should focus on prompt engineering—teaching students to craft high-yield prompts that elicit positive, mastery-affirming feedback from the bot, thereby helping create a virtuous cycle of confidence and practice. Second, to leverage the substantial direct and indirect influence of learning grit, interventions should move beyond short-term tasks to foster sustained persistence. Curricula could embed a semester-long GenAI dialogue project tied to high personal stakes, such as passing a crucial oral exam or preparing for a study-visa interview. Within this framework, instructors can require students to maintain bi-weekly reflection logs charting their persistence milestones and challenges. This approach frames language learning as a long-term process of mastery, providing tangible opportunities for grit to be developed, validated, and translated into sustained willingness to communicate.

#### 4.2.3 Limitations and future research

Despite these contributions, several limitations should be acknowledged. First, the study utilized self-reported data and a cross-sectional design, which, although informative regarding relationships among variables, cannot establish causality definitively. Future research should employ longitudinal or experimental designs to further investigate the causal pathways identified herein. Second, the participant sample was restricted to Chinese undergraduate students. This specific cultural context, characterized by a strong emphasis on “face” (*mianzi*) and a generally high acceptance of digital technology, may limit the generalizability of the findings. For instance, the anxiety-reducing effect of GenAI might be particularly pronounced in cultures where face-saving is a dominant social norm. Future studies should examine these relationships in diverse cultural settings to determine whether the proposed model holds true across different educational and cultural backgrounds. Third, regarding tool homogeneity, this study did not differentiate between specific interaction modalities (e.g., text-only chat versus voice-interactive mode). It is plausible that voice-based interaction elicits different levels of anxiety and enjoyment compared to text-based interaction. Future work should explicitly manipulate interface modality to rigorously test its impact on learners’ affective states and WTC. Fourth, a limitation regarding the data collection context must be noted. Since recruitment was facilitated by instructors within classroom settings, there is a potential risk of authority effects or social desirability bias. Although strict procedural measures were implemented to minimize these risks—specifically, ensuring that participation was anonymous, confidential, voluntary, and explicitly unrelated to academic grades—the institutional setting constitutes a factor that should be considered when interpreting the results. Finally, it would be valuable to examine additional variables, such as students’ digital literacy or the specific nature of GenAI tasks (e.g., conversational versus corrective), which may moderate observed relationships. Qualitative investigations could also yield deeper insights into the mechanisms underlying the influence of grit on WTC and the development of self-efficacy through successful AI interactions. As GenAI becomes increasingly integrated into educational environments, understanding these human factors will be essential. This study represents an important step toward that goal.

## Data Availability

The raw data supporting the conclusions of this article will be made available by the authors, without undue reservation.

## Appendix

**Table A1:** Summary of hypotheses testing and conclusions based on empirical results.

Hypothesis	Structural path	Supporting evidence	Outcome
H1	Learning Grit → Speaking Self-efficacy	*β* = 0.456, *p* < 0.001	Supported
H2	Speaking Self-efficacy → Speaking Anxiety	*β* = −0.425, *p* < 0.001	Supported
H3	Speaking Self-efficacy → Speaking Enjoyment	*β* = 0.465, *p* < 0.001	Supported
H4	Speaking Anxiety → WTC with GenAI	*β* = −0.142, *p* < 0.05	Supported
H5	Speaking Enjoyment → WTC with GenAI	*β* = 0.159, *p* < 0.05	Supported
H6	Speaking Self-efficacy → WTC with GenAI	*β* = 0.240, *p* < 0.001	Supported
H7	Learning Grit → WTC with GenAI	*β* = 0.232, *p* < 0.001	Supported

## References

[ref1] Alamer A. (2021). Grit and language learning: construct validation of L2-grit scale and its relation to later vocabulary knowledge. Educ. Psychol. 41, 544–562. doi: 10.1080/01443410.2020.1867076

[ref2] Aziz A. A. Kashinathan S. (2021). ESL learners’ challenges in speaking English in Malaysian classroom. Development 10, 983–991. doi: 10.6007/IJARPED/v10-i2/10355

[ref001] Bandura A. (1997). Self-efficacy: The exercise of control. W H Freeman/Times Books/ Henry Holt & Co.

[ref3] Bandura A. (2001). Social cognitive theory: an agentic perspective. Annu. Rev. Psychol. 52, 1–26. doi: 10.1146/annurev.psych.52.1.1 11148297

[ref4] Bárkányi Z. Brash B. (2025). Foreign language speaking anxiety online: mitigating strategies and speaking practices. ReCALL 37, 421–440.

[ref5] Bergil A. S. (2016). The influence of willingness to communicate on overall speaking skills among EFL learners. Procedia. Soc. Behav. Sci. 232, 177–187. doi: 10.1016/j.sbspro.2016.10.043

[ref6] Botes E. der Van Westhuizen L. Dewaele J. M. MacIntyre P. Greiff S. (2022). Validating the short-form foreign language classroom anxiety scale. Appl. Linguist. 43, 1006–1033. doi: 10.1093/applin/amac018

[ref7] Celik B. Yildiz Y. Kara S. (2025). Using ChatGPT as a virtual speaking tutor to boost EFL learners’ speaking self-efficacy. Aust. J. Appl. Linguist. 8:102418.

[ref8] Chen J. (2025). Examining the role of Chinese language learners' grit and self-efficacy on their engagement in artificial intelligence-driven settings. Acta Psychol. 259:105357. doi: 10.1016/j.actpsy.2025.105357 40768911

[ref9] Chen X. Alruwaili A. R. Azari Noughabi M. Ghasemi A. Zhen C. (2025). The mediating role of psychological capital in the relationship between EFL learners' L2 grit and L2 WTC. Front. Psychol. 16:1621340. 40709236 10.3389/fpsyg.2025.1621340 PMC12288664

[ref10] Deci E. L. Ryan R. M. 2012 Self-determination theory. In Lange P. A. M. Van Kruglanski A. W. Higgins E. T. (Eds.), Handbook of theories of social psychology, London: SAGE Publications Ltd. 1, 416–436.

[ref11] Du H. Guan H. (2016). Hindrances to the new teaching goals of college English in China: being contextually blind and linguistically groundless, current tertiary ELT policy needs to be redefined. Engl. Today 32, 12–17. doi: 10.1017/S0266078415000462

[ref12] Duckworth A. L. Peterson C. Matthews M. D. Kelly D. R. (2007). Grit: perseverance and passion for long-term goals. J. Pers. Soc. Psychol. 92, 1087–1101. doi: 10.1037/0022-3514.92.6.1087, 17547490

[ref13] Fathi J. Mohammaddokht F. Nourzadeh S. (2021). Grit and foreign language anxiety as predictors of willingness to communicate in the context of foreign language learning: a structural equation modeling approach. Issues Lang. Teach. 10, 1–30.

[ref14] Fernández-García A. Fonseca-Mora M. C. (2022). EFL learners’ speaking proficiency and its connection to emotional understanding, willingness to communicate and musical experience. Lang. Teach. Res. 26, 124–140. doi: 10.1177/1362168819891868

[ref15] Ghani M. Azhar S. W. (2017). Effect of motivation, willingness to communicate (WTC), self perceived communicative competence (SPCC) and L2 anxiety on the frequency of use of English as L2. J. Educ. Res. 20:157.

[ref16] Guo K. Wang D. (2024). To resist it or to embrace it? Examining ChatGPT’s potential to support teacher feedback in EFL writing. Educ. Inf. Technol. 29, 8435–8463. doi: 10.1007/s10639-023-12146-0

[ref17] Hair J. F. Black W. C. Babin B. J. Anderson R. E. (2010). Multivariate data analysis. 7th Edn. Upper Saddle River: Prentice Hall.

[ref18] Hargie O. (2021). Skilled interpersonal communication: Research, theory and practice. London: Routledge.

[ref19] Hu L. T. Bentler P. M. (1999). Cutoff criteria for fit indexes in covariance structure analysis: conventional criteria versus new alternatives. Struct. Equ. Model. Multidiscip. J. 6, 1–55. doi: 10.1080/10705519909540118

[ref20] Jin Y. Qin L. (2024). Examining Chinese university students’ English speaking enjoyment: scale development and validation. System 124:103382. doi: 10.1016/j.system.2024.103382

[ref21] Joe H. K. Hiver P. Al-Hoorie A. H. (2017). Classroom social climate, self-determined motivation, willingness to communicate, and achievement: a study of structural relationships in instructed second language settings. Learn. Individ. Differ. 53, 133–144. doi: 10.1016/j.lindif.2016.11.005

[ref22] Kang S. J. (2005). Dynamic emergence of situational willingness to communicate in a second language. System 33, 277–292. doi: 10.1016/j.system.2004.10.004

[ref23] Kline R. B. (2023). Principles and practice of structural equation modeling. New York: Guilford publications.

[ref24] Lee J. S. (2022). The role of grit and classroom enjoyment in EFL learners’ willingness to communicate. J. Multiling. Multicult. Dev. 43, 452–468. doi: 10.1080/01434632.2020.1746319

[ref25] Lee J. S. Hsieh J. C. (2019). Affective variables and willingness to communicate of EFL learners in in-class, out-of-class, and digital contexts. System 82, 63–73. doi: 10.1016/j.system.2019.03.002

[ref26] Leeming P. (2017). A longitudinal investigation into English speaking self-efficacy in a Japanese language classroom. Asian-Pac. J. Second. Foreign. Lang. Educ. 2:12. doi: 10.1186/s40862-017-0035-x

[ref27] Leeming P. Vitta J. P. Hiver P. Hicks D. McLean S. Nicklin C. (2024). Willingness to communicate, speaking self-efficacy, and perceived communicative competence as predictors of second language spoken task production. Lang. Learn. 74, 917–949. doi: 10.1111/lang.12640

[ref28] Li Z. Li B. Wang X. Zhen L. (2025). Clustering perceived learning environment as antecedents of willingness to communicate in a second language. Lang. Teach. Res. 29, 2777–2794. doi: 10.1177/13621688221122611

[ref29] MacIntyre P. D. Babin P. A. Clément R. (1999). Willingness to communicate: antecedents & consequences. Commun. Q. 47, 215–229. doi: 10.1080/01463379909370135

[ref30] MacIntyre P. D. Clément R. Dörnyei Z. Noels K. A. (1998). Conceptualizing willingness to communicate in a L2: a situational model of L2 confidence and affiliation. Mod. Lang. J. 82, 545–562. doi: 10.1111/j.1540-4781.1998.tb05543.x

[ref31] Maher K. King J. (2023). Language anxiety and learner silence in the classroom from a cognitive-behavioral perspective. Annu. Rev. Appl. Linguist. 43, 105–111. doi: 10.1017/S0267190523000077

[ref32] Mahmoodi M. H. Moazam I. (2014). Willingness to communicate (WTC) and L2 achievement: the case of Arabic language learners. Procedia. Soc. Behav. Sci. 98, 1069–1076. doi: 10.1016/j.sbspro.2014.03.518

[ref33] Nation I. S. (2008). Teaching ESL/EFL reading and writing. New York: Routledge.

[ref34] Newton J. M. Nation I. S. (2020). Teaching ESL/EFL listening and speaking. New York: Routledge.

[ref35] Pandey M. Pandey P. (2014). Better English for better employment opportunities. Int. J. Multidiscip. Approach Stud. 1, 93–100.

[ref36] Pawlak M. Mystkowska-Wiertelak A. (2015). Investigating the dynamic nature of L2 willingness to communicate. System 50, 1–9. doi: 10.1016/j.system.2015.02.001

[ref37] Peng J. E. Woodrow L. (2010). Willingness to communicate in English: a model in the Chinese EFL classroom context. Lang. Learn. 60, 834–876. doi: 10.1111/j.1467-9922.2010.00576.x

[ref38] Podsakoff P. M. MacKenzie S. B. Lee J. Y. Podsakoff N. P. (2003). Common method biases in behavioral research: a critical review of the literature and recommended remedies. J. Appl. Psychol. 88, 879–903. doi: 10.1037/0021-9010.88.5.879, 14516251

[ref39] Rahmatollahi M. Khalili G. F. (2015). Relationship between intermediate EFL learners' communication apprehension, willingness to communicate, and speaking ability. Int. J. App. Linguistics English Lit. 4:23. doi: 10.7575/aiac.ijalel.v.4n.6p.23

[ref40] Rauthmann J. F. Sherman R. A. Funder D. C. (2015). Principles of situation research: towards a better understanding of psychological situations. Eur. J. Personal. 29, 363–381. doi: 10.1002/per.1994

[ref41] Riasati M. J. Noordin N. (2011). Antecedents of willingness to communicate: a review of literature. Stud. Lit. Lang. 3:74.

[ref42] Ryan R. M. Deci E. L. (2002). Overview of self-determination theory: an organismic dialectical perspective. In Deci E. L. Ryan R. M. (Eds.) Handbook of self-determination research, Rochester, NY: University of Rochester Press. 2, 36.

[ref43] Salbaş H. Ekmekci E. (2025). The impact of classroom environment on students' willingness to communicate in foreign language learning. Int. J. Educ. Res. 129:102517. doi: 10.1016/j.ijer.2024.102517

[ref44] Suleimenova Z. (2013). Speaking anxiety in a foreign language classroom in Kazakhstan. Procedia Soc. Behav. Sci. 93, 1860–1868. doi: 10.1016/j.sbspro.2013.10.131

[ref45] Teimouri Y. Goetze J. Plonsky L. (2019). Second language anxiety and achievement: a meta-analysis. Stud. Second. Lang. Acquis. 41, 363–387. doi: 10.1017/S0272263118000311

[ref46] Vygotsky L. S. (1978). Mind in society: The development of higher mental processes. Cambridge: Harvard University Press.

[ref47] Wang W. Sabbaghi P. Izadpanah S. (2023). The study of the relationship between willingness to communicate and self-regulation with the mediating role of self-efficacy among English foreign language learners: structural equation modelling approach. Curr. Psychol. 44, 1–16.

[ref48] Wang L. Wang L. (2025). Exploring enjoyment, motivation, self-efficacy, and engagement in AI-assisted English learning: a self-determination theory approach. Learn. Motiv. 92:102197. doi: 10.1016/j.lmot.2025.102197

[ref49] Wu T. T. Hapsari I. P. Huang Y. M. (2025). Effects of incorporating AI chatbots into think–pair–share activities on EFL speaking anxiety, language enjoyment, and speaking performance. Comput. Assist. Lang. Learn., 1–39.

[ref50] Zarrinabadi N. Lou N. M. Darvishnezhad Z. (2023). To praise or not to praise? Examining the effects of ability vs. effort praise on speaking anxiety and willingness to communicate in EFL classrooms. Innov. Lang. Learn. Teach. 17, 88–101. doi: 10.1080/17501229.2021.1938079

[ref51] Zhang J. Beckmann N. Beckmann J. F. (2018). To talk or not to talk: a review of situational antecedents of willingness to communicate in the second language classroom. System 72, 226–239. doi: 10.1016/j.system.2018.01.003

[ref52] Zhang C. Meng Y. Ma X. (2024). Artificial intelligence in EFL speaking: impact on enjoyment, anxiety, and willingness to communicate. System 121:103259. doi: 10.1016/j.system.2024.103259

[ref53] Zhang Q. Nie H. Fan J. Liu H. (2025). Exploring the dynamics of artificial intelligence literacy on English as a foreign language learners’ willingness to communicate: the critical mediating roles of artificial intelligence learning self-efficacy and classroom anxiety. Behav. Sci. 15:523. doi: 10.3390/bs15040523 40282144 PMC12024140

[ref55] Zhou L. Xi Y. Lochtman K. (2023). The relationship between second language competence and willingness to communicate: the moderating effect of foreign language anxiety. J. Multiling. Multicult. Dev. 44, 129–143. doi: 10.1080/01434632.2020.1801697

